# Changing educational attainment as a driver of cohort changes in healthy longevity: a decomposition analysis of US birth cohorts

**DOI:** 10.1093/aje/kwaf066

**Published:** 2025-03-27

**Authors:** Tianyu Shen, Alyson van Raalte, Collin F Payne

**Affiliations:** Vienna Institute of Demography, Austrian Academy of Sciences, Vienna, Austria; Max Planck—University of Helsinki Center for Social Inequalities in Population Health (MaxHel Center), Rostock, Germany and Helsinki, Finland; School of Demography, Australian National University, Canberra, Australia; Harvard Center for Population and Development Studies, Harvard T.H. Chan School of Public Health, Cambridge, United States

**Keywords:** disability-free life expectancy, decomposition, education composition, multistate life table, health and aging

## Abstract

An anticipated health boost from the increasing educational attainment of the US population has not materialized, with life expectancy and healthy longevity both stagnating over the past decade. We seek to understand how changes in the level of educational attainment across successive birth cohorts in the United States have impacted disability-free life expectancy (DFLE) among older Americans. We analyze data from the US Health and Retirement Study spanning 2000 to 2020, focusing on four consecutive 10-year birth cohorts. We then decompose changes in population-level expectancies into contributions from shifts in educational composition, health status at midlife, and health and mortality transitions at older ages across different educational groups. Disability-free life expectancy increased notably for females but not for males, with disabled life expectancy (DLE) remaining stable. Shifts in educational composition primarily drove increases in DFLE and total life expectancy. However, deteriorating midlife health among those without a high school diploma reduced DFLE for this group, which tempered overall population-level gains. Health and mortality transitions among the less educated contributed to increased DLE. Our findings show that educational attainment is a major structural factor influencing the US population’s health. Expanding access to higher education and reducing education inequality will play a significant role in future changes to healthy longevity.

## Introduction

Following a century or more of substantial growth, life expectancy (LE) in the United States stagnated over the past decade, before declining during the COVID-19 pandemic years.^1^ This stagnation in LE can be partly explained by rising mortality among low-educated Americans, which offsets increases in LE among the college-educated.^2^^,^^3^ Similarly, while improvements in disability-free LE (DFLE) were observed from the 1980s up to the early 2000s,^2^^,^^4^^,^^5^ more recent research finds that advances in DFLE have plateaued since the turn of the new century.^6^^,^^7^ The expected lifetime with disability has also seen very little compression across cohorts.^8^

These dismal trends are in some ways surprising, given that the older US population is increasingly more educated, and education is consistently associated with better health.^9^ An anticipated health boost from population-compositional changes in education^9^ has not materialized. As one of the first countries to undergo educational expansion,^10^ the older US population is expected to be at the forefront of educational–compositional health boosts compared to other countries. Contrary to this development, trends in LE and DFLE in the United States have stagnated in the most recent decade,^7^^,^^8^^,^^11^ and the United States is falling further behind peer countries.^12^^,^^13^

Studying population-level changes in LE and DFLE is crucial for tracking health across the nation. Extensive prior research has reported these indices by sex, suggesting that women live longer but in worse health.^14^^,^^15^ However, other socioeconomic factors heavily influence health and well-being at later ages. Link and Phelan^16^ consider these factors as “fundamental causes” of disparities in health and longevity as they are related to individuals’ risk factors and access to resources that can shape disease outcomes. Education is one of the most important socioeconomic factors impacting disparities in health and mortality.^3^^,^^9^^,^^17^ Lutz et al.^18^^,^^19^ argue that educational attainment should be considered the third key dimension in demographic analysis, alongside age and sex.

A review by Hayward et al.^20^ identified 4 key mechanisms through which education directly impacts adult health: (1) material pathways including improved employment opportunities, higher income, safer working conditions, and resilience to economic crises; (2) knowledge and health literacy to adopt healthy lifestyles and access health care; (3) access to social networks to capitalize on technologically advanced treatments; and (4) cognitive skills, greater sense of control, and human agency. These multifaceted impacts of education on adult health highlight the role of education as among the most important axes of social stratification. Studies have also shown that the functional form relating education to mortality and health could differ by sex.^21^ Resource substitution and human capital perspectives suggest a stronger role of education on women’s health than men’s in contexts where women possess fewer socioeconomic resources, making them more dependent on education for well-being or physical health.^22^^,^^23^ However, there are mixed results on the gender differential depending on the health outcomes and studied populations.^24^^,^^25^

Most research on educational inequalities in health and mortality focuses on measuring differences in health and mortality between educational groups and how these between-group differences have changed over time. Prior research has found that LE for the least educated group appears to decline over time and that inequalities are widening.^26^^‑^^28^ These studies posit that growing educational inequality may be contributing to the recent stagnation seen in US mortality. However, these changes are occurring while the educational composition of the US population is experiencing a massive shift towards increased educational attainment. According to the US Census Bureau,^29^^,^^30^ the proportion of the US population with a bachelor’s degree between the ages of 30 and 59 rose from 31.5% in 2010 to 40.1% in 2020, while the proportion without a high school diploma dropped from 11.1% to 8.6%.

Focusing only on between-group comparisons over time largely fails to consider these compositional changes that, at the population level, should have improved health. Prior research has investigated the effect of education structure on differences in period LE.^31^^,^^32^ However, we need to understand how these changes in the educational structure of the population contribute to overall population health longevity, rather than focusing solely on mortality as an outcome. Exploring health expectancies provides more nuanced insight into how the concurrent processes of educational inequality in health and the changing composition of educational subgroups connect to population-level health and wellbeing.

To some extent, whether we expect a large gain in health at the population level from educational expansion comes down to the role played by education in promoting health. Credentialist arguments hold that each additional person-year of education in the population brings tangible benefits such as health literacy and health promotion.^33^ In this view, education promotion is a health policy. Relativist views, on the other hand, argue that the higher educated are generally in better health because of their higher position in a social hierarchy, and lower psychosocial stress.^34^^,^^35^ Empirically, it is challenging to separate these mechanisms that are likely to operate in parallel.

Additionally, most present research focuses on health at older ages but overlooks the cumulative health effects from younger ages. Studies examining remaining life and health expectancies at older ages often estimate these measures starting at ages after 50. However, the population health composition at these older ages is a key determinant of health expectancies in later life, and this composition results from an accumulation of trends from earlier in life. Thus, it is important to understand how much of the change in healthy LE among older populations results from health outcomes accumulated from younger ages.

This paper aims to explore how changes in population-level educational composition and changes in health within different educational groups have combined to lead to changes in healthy longevity in the US population. Our analyses decompose the drivers of cohort differences in population-level DFLE into 3 distinct components: (1) the portion contributed by changes in educational composition (*P-effect*); (2) the portion resulting from educational differences in health at the baseline age (*I-effect*); and (3) the portion driven by changes in patterns of mortality and onset/recovery from disability across educational groups (*H-effect*). These analyses shed new light on how population-level educational composition and changes in the education–health relationship over cohorts have impacted population health in the United States.

## Methods

### Data and measures

Our data are from the longitudinal US Health and Retirement Survey (HRS), a nationally representative biennial longitudinal survey of the US population aged 51 and older.^36^ We use one of the most common indicators, disability, to serve as a proxy for health. Disability is defined by 5 activities of daily living (ADLs). If the respondents reported difficulties in bathing, dressing, eating, transferring in/out of bed, or walking across a room, they are classified as disabled (D); otherwise, they are disability-free (DF). Educational attainment is classified into 4 categories: (1) less than a high school diploma (<HS), (2) high school graduate, including GED (HS), (3) some college (Col.), and (4) bachelor’s degree or higher (Bac.). Our mortality data are provided by HRS, which reliably records deaths by tracking respondents’ survival through its own efforts and linkages to the National Death Index.^37^ A recent comparison of several US survey–linked mortality records (including HRS) and vital statistics data found reassuringly consistent mortality hazard ratios of key sociodemographic variables such as sex, race, and education.^38^

Our analyses focus on measuring and comparing cohort life and health expectancies, as the process of educational expansion is one that evolves over successive cohorts. Unlike period life expectancy, which is based on a synthetic aggregation of multiple cohorts, these cohort measures of life expectancy avoid introducing bias due to temporal shift in educational composition across birth cohorts,^7^ providing a clearer view of changes in healthy longevity tied to specific population dynamics. This approach allows us to examine how changes in educational composition impact the lifespan of birth cohorts in the population, as our results directly capture the lived experiences of actual individuals within educational groups, rather than representing an aggregate measure at a point in time. However, a full cohort analysis requires following up on the extinct cohort of individuals for a duration of 100 years. To assess changes across birth cohorts of living individuals, we estimate partial cohort life expectancy over a 10-year span using data from 2000 (wave 5) to 2020 (wave 15) (we start from wave 5 because HRS provides sample weights for individuals in nursing homes onward). These 10-year partial cohort expectancies could reach a maximum of 10 years should all individuals survive the full period.

**Figure 1:**
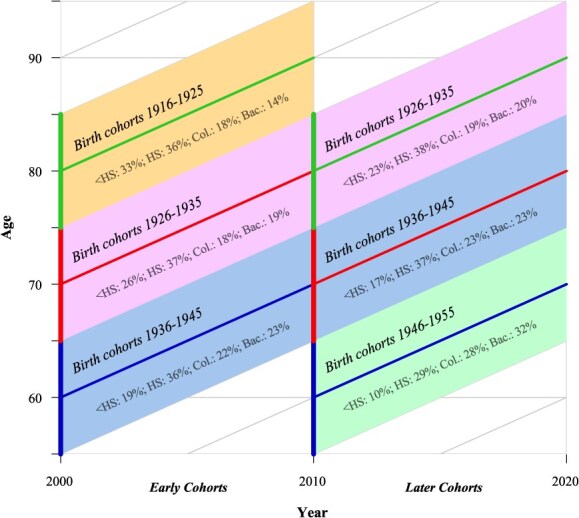
Lexis diagram of the study design: birth cohorts are followed for 10 years and compared to same-age birth cohorts born 10 years later. The percentages of each educational subgroup at the start of the age interval are given for each 10-year birth cohort (Bac., bachelor’s degree or higher; Col. = some college; <HS, below high school; HS, high school). Authors’ calculations based on HRS.^22^

This study compares health expectancies in ages 60-69, 70-79, and 80-89 across successive 10-year birth cohorts (1916-1925, 1926-1935, 1936-1945, and 1946-1955). Figure 1 shows the corresponding birth cohorts observed within a specific 10-year period, with “early cohorts” on the left and “later cohorts” on the right for each age range. All individuals from these birth cohorts with at least 2 consecutive observations of disability status or mortality are included, excluding cases missing education or gender (see Table S1 for sample sizes). We show the proportions of each educational subgroup at the start of the age range below the birth cohorts in Figure 1. Table S2 presents the sample characteristics of these birth cohorts at baseline (observed at wave 5 or wave 10). The least educated group shrinks across successive cohorts in all age ranges, while the most educated group expands. Both sexes experience similar percentage expansions over time.

### Analyses

Transition probabilities are estimated using a multinomial logistic regression model, incorporating disability states by age, sex, education, age-squared, and interactions between sex and age or education.^8^ The number of observed transitions between health states for each birth cohort is presented in Table S3. Sample weights (respondent and nursing home resident weights) from the HRS at each wave are applied. We bootstrap the HRS data 500 times to obtain CIs.

After obtaining the baseline health structure and the transition probabilities from the regression, partial cohort life expectancy from age $\alpha$ to age $\beta$ can be calculated as


(1) \begin{equation*} {}_{\beta -\alpha }{}{\mathbf{e}}_{\alpha }=\frac{{\mathbf{l}}_{\alpha }}{2}+\sum_{x=\alpha +1}^{\beta -1}{\mathbf{l}}_x+\frac{{\mathbf{l}}_{\beta }}{2} \end{equation*}

where ${\mathbf{l}}_x$ is the age-specific survival matrix computed from the product of transition matrix, ${\mathbf{P}}_x$, with the initial health distribution of the survivor at age $\alpha$, ${\mathbf{l}}_x={\mathbf{l}}_{\mathrm{\alpha}}\prod_{k=\mathrm{\alpha}}^{x-1}{\mathbf{P}}_k$.^39^ In this paper, we extend this equation by including educational subgroups. Since our study involves cohorts of elderly individuals, it is safe to assume that no migration occurs and that no participant changes their educational attainment level at these advanced ages. With the assumptions, the relationship between the components of each subpopulation and the partial life expectancy from age $\alpha$ to age $\beta$ of the total population, ${}_{\beta -\alpha }{}{\mathbf{e}}_{\alpha }$, in a closed population setting can be expressed as


(2) \begin{equation*} {\displaystyle \begin{array}{c}{}_{\beta -\alpha }{}{\mathbf{e}}_{\alpha }=\sum_k{c}_{\alpha}^k\left(\frac{{\mathbf{l}}_{\alpha}^k}{2}+\displaystyle\sum_{x=\alpha +1}^{\beta -1}{\mathbf{l}}_x^k+\frac{{\mathbf{l}}_{\beta}^k}{2}\right)\end{array}} \end{equation*}

where ${c}_{\alpha}^k$ is the population proportion of the educational subgroup, $k$, at age $\alpha$ when the subnational populations are exclusive and individuals rarely move between subgroups from $\alpha$ to $\beta$, and ${\mathbf{l}}_x^k$ is the survival matrix of subnational population, $k$*,* at age $x$. To test the sensitivity of the assumptions, Table S4 displays 2 sets of life expectancy calculations: Panel A shows life expectancies for the total population without considering educational groups [eqn (1)], while Panel B shows life expectancies calculated using eqn (2) with the educational groups. The results from these 2 approaches are nearly identical.

Shen et al.^39^ decompose the change in multistate life expectancies based on eqn (1) (represented as the Newton derivative dot on top of the variable) into the contribution from the initial population health distribution and the contribution from transition probabilities



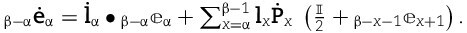



Following a similar procedure, the change over time of the multistate temporary life expectancy of eqn (2) can be calculated as


(3)

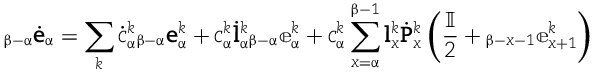



where 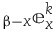 is the status-based life expectancy of the sub-population, $k$, between ages $x$ and $\beta$, and 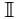 is the identity matrix. Thus, the change in partial life and health expectancies of the total population is decomposed into 3 components in each sub-population: (1) the effect of the change in the educational composition across the 2 cohorts (*P-effect*); (2) the effect from the change in health composition at the baseline age across the 2 cohorts (*I-effect*); and (3) the effect from the change in health and mortality transitions at older ages across the 2 cohorts (*H-effect*, the last term, 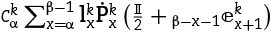, in eqn (3).

## Results

### Health expectancies by cohort and age

**Figure 2:**
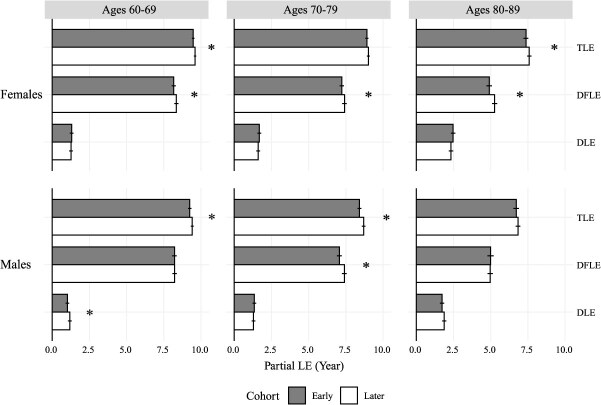
Partial cohort life expectancy between 2 cohorts by sex and age range. All expectancies include 95% CI. Asterisk indicates a significant difference between the 2 cohorts (found also in Table 1). DFLE, 10-year partial disability-free life expectancy; DLE, 10-year partial disabled life expectancy; TLE, 10-year partial total life expectancy. Authors’ calculations based on HRS.^22^

Figure 2 illustrates partial cohort DFLE and disabled life expectancy (DLE) by sex, birth cohort, and age group. Ninety-five percent CIs are included as error bars. The sum of DFLE and DLE is total life expectancy (TLE). Males have lower TLE than females across all age groups, and TLEs increase across cohorts, aligning with the results of other studies.^22^^,^^40^ By comparing the later cohorts (white bars) with the early ones (gray bars) in Figure 2, we see that the improvement in DFLE across cohorts is larger for females but still relatively small across all age groups, while males experience little change in DFLE but increases in DLE. The significance of the difference is indicated by stars on the right.

### Decomposing changes across cohorts

**Figure 3:**
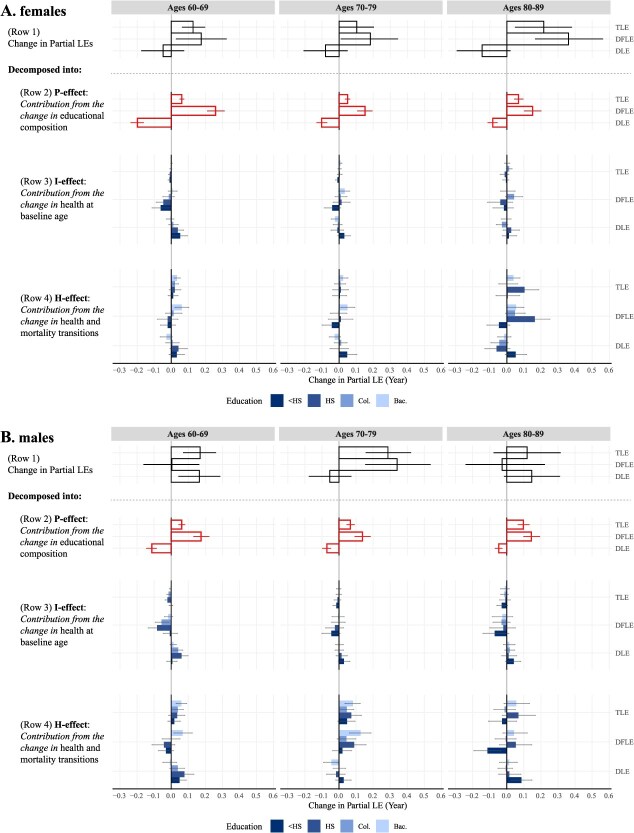
Ten-year change in partial cohort life expectancy between 2 cohorts (Later to Early) and contributions from different components by educational attainment. (A) Females. (B) Males. 95% CI is included, significant if not crossing zero. The P-effect bars are aggregated across all education groups. Bac., bachelor’s degree or higher; Col., some college; DFLE, 10-year partial disability-free life expectancy; DLE, 10-year partial disabled life expectancy; <HS, below high school; HS, high school; TLE, 10-year partial total life expectancy. Authors’ calculations based on HRS.^22^

Row 1 of Figure 3 presents the change between 10-year cohorts by age range for females (panel A) and males (panel B), which is the gap between the early and later cohorts in Figure 2. Applying eqn (3), these changes in Row 1 are decomposed into the education-specific contributions from 3 components: P-effect (Row 2), I-effect (Row 3), and H-effect (Row 4). The P-effect is aggregated across educational groups to show the total contribution of educational compositional change (in red bars), while the I-effect and H-effect are separated by educational groups. Table 1 shows the total contribution from each of the components aggregated by all educational groups. The P-effect corresponds to the values of the red bars in Figure 3 (Row 2) in Table 1, while I-effect and H-effect are the sums of the blue bars in Figure 3. The sum of these 3 effects is the total cohort change in the 10-year partial life expectancies. The values are significant if the error bars in Figure 3 do not cross zero and are marked with an asterisk in Table 1.

**Table 1:** Contribution from different components to the 10-year cohort change in partial life expectancies.

**Age** **(cohorts)**	**Component**	**Females**			**Males**		
		**DFLE**	**DLE**	**TLE**	**DFLE**	**DLE**	**TLE**
60-69 (1946-1955 to 1936-1945)	**P-effect** Population structure	0.26^*^	−0.20^*^	0.06^*^	0.17^*^	−0.11^*^	0.06^*^
	**I-effect** Health at baseline age	−0.12^*^	0.10^*^	−0.02^*^	−0.16^*^	0.12^*^	−0.04^*^
	**H-effect** Health and mortality transitions	0.03	0.05	0.09^*^	−0.01	0.16^*^	0.15^*^
	Total change	0.18^*^	−0.05	0.13^*^	0.00	0.17^*^	0.17^*^
70-79 (1936-1945 to 1926-1935)	**P-effect** Population structure	0.15^*^	−0.10^*^	0.05^*^	0.14^*^	−0.07^*^	0.07^*^
	**I-effect** Health at baseline age	0.02	−0.01	0.01	−0.08	0.05	−0.03
	**H-effect** Health and mortality transitions	0.01	0.03	0.05	0.28^*^	−0.03	0.25^*^
	Total change	0.18^*^	−0.08	0.10	0.34^*^	−0.05	0.29^*^
80-89 (1926-1935 to 1916-1925)	**P-effect** Population structure	0.15^*^	−0.08^*^	0.07^*^	0.14^*^	−0.05^*^	0.10^*^
	**I-effect** Health at baseline age	−0.01	0.01	0.00	−0.15^*^	0.08^*^	−0.06^*^
	**H-effect** Health and mortality transitions	0.22^*^	−0.07	0.15	−0.02	0.11	0.08
	Total change	0.36^*^	−0.14	0.22^*^	−0.03	0.15	0.12

Abbreviations: DFLE, 10-year partial disability-free life expectancy; DLE, 10-year partial disabled life expectancy; TLE, 10-year partial total life expectancy.

Values with asterisk are significant at 95% CIs.

Authors’ calculation based on HRS.^22^

### P-effect: population educational composition

This component represents the changes in LE and DFLE contributed by shifts in educational structure over cohorts and is shown in Row 2 of Figure 3. Both sexes exhibit similar tendencies. This component is more intuitive as a total effect without disaggregating by education because the changes in different groups are interrelated and constrained. Therefore, we present the net impact after summing up the contribution from shifts in educational group size, represented in the red bars. Changes in educational composition increased DFLE for women by 0.26, 0.15, and 0.15 years for this 10-year partial life expectancy at ages 60-69, 70-79, and 80-89. For men, changing education composition led to even greater increases in DFLE, with gains of 0.17, 0.14, and 0.14 years for each decade. At the same time, these compositional changes resulted in relatively smaller reductions in DLE: −0.20, −0.10, and −0.08 years for women and −0.11, −0.07, and −0.05 years for men across the same age groups. The shift in educational composition seems to reduce DLE slightly more for females than for males. In short, net of the impact from changing health and mortality dynamics, the compositional changes in education across cohorts have acted to raise DFLE and decrease DLE, contributing to a compression of disability over cohorts and an increase in total life expectancy.

### I-effect: changes in health at baseline age

The second component, the health at baseline age (I-effect), is measured by the proportion of people disabled or DF at the age of baseline for each education category. This component can also be understood as the contribution of cumulative health before the baseline age. The results suggest that, within most educational groups, the baseline disability status of cohorts at ages 60, 70, and 80 is not improving DFLE across cohorts, especially for individuals with a high school diploma or less. Gradients between education and disability can be seen across all age groups. The least negative, if not slightly positive, contribution to DFLE is typically made by those with a bachelor’s degree or higher. As shown in Table 1, the total contribution from changes in health at baseline to the 10-year partial DFLE is −0.12, 0.02, and −0.01 years for women and −0.16, −0.08, and −0.15 years for men at ages 60-69, 70-79, and 80-89. In contrast, holding constant the education structure and transition probabilities, the shift in health at baseline has acted to increase DLE for both women and men. This pattern is more pronounced at ages 60-69 and is stronger for males than females, though the overall effect of this component remains relatively minor.

### H-effect: changes in health and mortality transitions

The last component contributing to the change in DFLE and DLE across cohorts, the H-effect, represents changes in the disability and mortality transitions within a given age range (Row 4 of Figure 3). We find heterogeneity in the contribution of these transitions by education, with the change in disability transitions among the highly educated group contributing to a positive and significant increase in DFLE, while among the lowest educated group, they seem to contribute somewhat negatively. The less educated groups are likely to experience an expansion in disability due to the shifts in these transitions. The contributions to DFLE and DLE from disability and mortality transitions lead to an increase in TLE in almost all age-, sex-, and educational groups, though these are mostly non-significant apart from the most educated group. Combined, the H-effect results suggest that changes in disability and mortality transitions aggregated across education have generally acted to increase TLE (Table 1). For example, the H-effect increases the 10-year partial TLE at ages 60-69 by 0.09 years for females and 0.15 years for males due to cohort changes. At the population level, in Table 1, these transitions do not generally lead to a reduction in DLE, mainly due to the positive contribution to DLE from the less educated groups.

## Discussion

Changes in the educational distribution over cohorts are having a major impact on DF and disabled life years, despite what seems like stagnant or only slowly improving trends at the population level. In the absence of this compositional change, trends in DFLE would look much worse over cohorts. The older adult population will continue to be highly affected by these educational composition changes as successive cohorts reach older ages with higher overall levels of educational attainment.^29^^,^^30^ Educational expansion will play a significant, and potentially growing, role in future changes in life expectancy and healthy longevity.

In prior research, trends in health by education are typically examined within separate groups. Most of these studies also show worsening outcomes for those with lower levels of education and an improvement in DFLE for the most educated group.^7^^,^^26^^,^^41^ However, by ignoring the role of compositional changes in the population, this research has not fully explored the overall impact of education on population-level life and health expectancies. Thus, this study quantifies how changes in educational composition and changes occurring within educational sub-groups combine to shape national-level changes in healthy longevity. Our decomposition analysis represents a step forward in identifying the subpopulation factors behind changes in population life expectancy and healthy life expectancy.

Although changes in the educational composition across cohorts have contributed to improvements in DFLE, our findings suggest that successive cohorts have not experienced substantial improvements in health at baseline (cumulative health at younger ages). While survivorship to the ages we considere here (60 and above) has increased across cohorts,^42^^,^^43^ individuals were more likely to arrive at older ages with functional disabilities. This pattern is found most strongly among the lower-educated groups in our study. The cumulative disability component of our decomposition stresses that health in younger ages is very much relevant in older ages. Studies have shown that working-age adult mortality is rising in the United States, particularly among the least educated.^27^^,^^44^ However, as this paper emphasizes, it is crucial to look beyond mortality and consider other dimensions of health, such as disability, which have received less attention.^45^^,^^46^ Future health research and public health policies should focus on addressing inequalities in various health aspects among lower-educated groups from younger ages. When looking at changes in DFLE at older ages for this group (ages 70-79 or 80-89) the changing disability status at the start of the age interval was comparatively less important. This pattern may be explained by the age-as-leveler hypotheses arising from mortality selection.^43^^,^^47^

Our results found that changes in health transitions at older ages do little to compress disability, though they may increase the lifetime spent free of disability over cohorts depending on the age group and sex. An education-health gradient can be seen in the changes in health transitions. If anything, the least educated group tends to see an expansion of DLE. This is in line with research^26^^,^^48^ proposing that various contextual factors that have eroded the resources necessary for less educated persons to maintain good health despite an advancement of life-saving medicine that has kept them alive for longer.

Several prior studies also suggest that the pattern of selection into different levels of education varies over cohort.^17^^,^^41^^,^^49^^,^^50^ This process may leave a highly selected group of the most disadvantaged individuals in the lowest educated group. A missing piece in this argument, however, is the composition of the other educational groups as individuals are negatively selected into the least educated groups. Put another way, changes in educational selection across cohorts should also be observed in the highest education group, where more disadvantaged individuals were able to achieve higher levels of schooling in later cohorts. If selection was purely the driver of the differences we see, we would expect that the transition probabilities would also lead to worsening disability conditions in the higher educated groups as the size of these groups increased substantially, thus becoming less exclusive or health-positively selected, over cohorts. Yet, what we find is that the health transitions for individuals with a bachelor’s degree acted to improve DFLE. This suggests that selection is unlikely to be the sole factor at work.

A final thread of studies^26^^,^^48^^,^^51^^,^^52^ argues that educational expansion has itself caused changes in society that lead less educated individuals to face greater inequities throughout their life course. In contrast, the more educated individuals may benefit from this shift as educational qualifications have become more important for employment and social connections.

Although neither this study nor the prior studies offer conclusive evidence of this cause of the change, our findings nonetheless reveal that education is a large and growing marker of social disparity. Regardless of whether educational inequalities are driven by compositional change, materialist, or psychosocial factors, the fact that educational-based health disparities have widened is undeniable. The contribution from the health transitions shows that, unlike for the lower education groups, the improved health of higher education groups over cohorts appears to overcome the reduced selectivity of these groups. These changes in educational composition have occurred alongside societal changes that have increased the valuation of education, leading to increased inequality. Even in the absence of further changes from selectivity, these disparities are unlikely to narrow in the future unless earlier interventions and better health supports are available to the less educated groups.

Educational attainment is deeply integrated into modern society and serves as a broader representation of inequality, but it is certainly not the only source of health inequalities. One limitation of this study is the lack of analysis on potential heterogeneity in the contribution of education across, for instance, different race/ethnicity groups. Research suggests that the relationship between education and mortality may vary among these groups,^20^ which could also provide insights into disparities in health. However, the sample size of several race–education strata was too small to draw meaningful conclusions from this methodology. Even limiting the analysis to Black and White races resulted in several age–cohort–sex–education combinations with fewer than 15 individuals. While this study does not explore these interactions, we acknowledge this as a limitation, highlighting it as an area for future research. Additionally, the multistate model used is a Markov model, which assumes that future health states depend only on the current state, not the past trajectory. This assumption is common in multistate methods and unlikely to affect DFLE estimates.^53^ Another limitation arises from the biannual nature of HRS data, which assumes only 1 transition between waves, potentially overlooking back-and-forth transitions or health deterioration before death—a limitation inherent to the data collection process.^54^ Finally, this study defines health solely in terms of ADL disability, whereas other health measures and dimensions warrant further investigation in future research.

### Conclusions

We find that the modest gains in LE and DFLE across cohorts in the United States result almost entirely from shifts in the distribution of educational attainment, underpinned by growing inequality between educational groups. Improvements in the health and survival of the more educated are being largely offset by deteriorating trends among the less educated at older ages. Worsening trends in cumulative health outcomes up to midlife among those without a high school diploma contribute to a decline in DFLE both for this group and the entire US population. Continued increases in access to higher education will play a significant role in future changes to healthy longevity. More importantly, urgent action is needed to address health disparities that are often disguised by the stagnation in the overall population health.

## Supplementary Material

Web_Material_kwaf066

## Data Availability

HRS data are publicly available at: https://hrsdata.isr.umich.edu/data-products.
