# Supplementary material for: Changing educational attainment as a driver of cohort changes in healthy longevity: a decomposition analysis of US birth cohorts
Source: Am J Epidemiol. 2025 Mar 27;195(5):1222–30. doi: 10.1093/aje/kwaf066 (PMC13149017; doi:10.1093/aje/kwaf066)
Supplement: Web_Material_kwaf066 [file web_material_kwaf066.docx]

Changing educational attainment as a driver of cohort changes in healthy longevity: a decomposition analysis of US birth cohorts

Tianyu Shen, Alyson Van Raalte, Collin F. Payne

**Supplementary Material**

Supplementary Tables S1-S4

**Supplementary Table S1. Sample inclusion**

| **Age** | **Sample** | **Early cohorts** | **Later cohorts** |
| --- | --- | --- | --- |
| **60-69** | *Birth cohort* | *1936-1945* | *1946-1955* |
|  | All surviving individuals at start | 7,269 | 7,050 |
|  | Individuals without missing education or gender information | 7,266 | 7,049 |
|  | Individuals with two or more consecutive observations | 7,036 | 6,874 |
| **70-79** | *Birth Cohort* | *1926-1935* | *1936-1945* |
|  | All surviving individuals at start | 6,194 | 5,759 |
|  | Individuals without missing education or gender information | 6,193 | 5,757 |
|  | Individual with two or more consecutive observations | 6,044 | 5,720 |
| **80-89** | *Birth Cohort* | *1916-1925* | *1926-1935* |
|  | All surviving individuals at start | 4,051 | 4,093 |
|  | Individuals without missing education or gender information | 4,051 | 4,092 |
|  | Individual with two or more consecutive observations | 3,971 | 4,073 |

**Supplementary Table S2.** Sample characteristics of the birth cohort at baseline by sex

|  | Sex | **Females** | | **Males** | |
| --- | --- | --- | --- | --- | --- |
|  |  | **Early** | **Later** | **Early** | **Later** |
| **60** | *Birth Cohort* | *1936-1945* | *1946-1955* | *1936-1945* | *1946-1955* |
|  | N | 3,496 | 3,609 | 2,748 | 2,754 |
|  | ADL disabled (%) | 12.8 | 13.2 | 10.2 | 13.0 |
|  | Education (%) |  |  |  |  |
|  | *<HS* | 20.1 | 10.5 | 17.8 | 10.0 |
|  | *HS* | 38.5 | 30.9 | 34.0 | 26.6 |
|  | *Col.* | 23.4 | 28.1 | 20.6 | 28.7 |
|  | *Bac.* | 18.1 | 30.4 | 27.5 | 34.7 |
|  | Race (%) |  |  |  |  |
|  | *White* | 78.8 | 75.5 | 80.8 | 77.1 |
|  | *Black* | 10.6 | 12.0 | 9.6 | 10.7 |
|  | *Hispanic* | 8.3 | 8.6 | 7.3 | 8.4 |
|  | *Other* | 2.3 | 3.9 | 2.4 | 3.7 |
| **70** | *Birth Cohort* | *1926-1935* | *1936-1945* | *1926-1935* | *1936-1945* |
|  | N | 2,929 | 3,144 | 2,403 | 2,446 |
|  | ADL disabled (%) | 16.3 | 14.5 | 12.4 | 13.2 |
|  | Education (%) |  |  |  |  |
|  | *<HS* | 25.6 | 18.3 | 26.3 | 16.4 |
|  | *HS* | 41.0 | 38.6 | 32.2 | 34.5 |
|  | *Col.* | 18.3 | 24.0 | 17.2 | 21.0 |
|  | *Bac.* | 15.1 | 19.1 | 24.3 | 28.1 |
|  | Race (%) |  |  |  |  |
|  | *White* | 81.8 | 79.7 | 84.0 | 81.0 |
|  | *Black* | 10.3 | 10.0 | 8.1 | 8.2 |
|  | *Hispanic* | 5.7 | 8.2 | 5.7 | 8.4 |
|  | *Other* | 2.2 | 2.1 | 2.2 | 2.3 |
| **80** | *Birth Cohort* | *1916-1925* | *1926-1935* | *1916-1925* | *1926-1935* |
|  | N | 2,116 | 2,288 | 1,470 | 1,746 |
|  | ADL disabled (%) | 26.6 | 25.4 | 20.7 | 23.0 |
|  | Education (%) |  |  |  |  |
|  | *<HS* | 31.6 | 22.1 | 33.8 | 23.6 |
|  | *HS* | 39.0 | 41.8 | 31.1 | 33.0 |
|  | *Col.* | 18.9 | 20.5 | 16.3 | 17.1 |
|  | *Bac.* | 10.5 | 15.6 | 18.7 | 26.4 |
|  | Race (%) |  |  |  |  |
|  | *White* | 87.0 | 82.5 | 86.8 | 84.7 |
|  | *Black* | 7.2 | 8.9 | 6.6 | 7.5 |
|  | *Hispanic* | 4.2 | 6.3 | 4.8 | 5.5 |
|  | *Other* | 1.5 | 2.3 | 1.8 | 2.3 |

**Supplementary Table S3.** Number of transitions by birth cohort

| **Age** | **Time** $\boldsymbol{t}$ | **Time** $\boldsymbol{t+1}$ | | | **Time** $\boldsymbol{t+1}$ | | |
| --- | --- | --- | --- | --- | --- | --- | --- |
|  |  | **DF** | **D** | **Dead** | **DF** | **D** | **Dead** |
| **60-69** | *Birth cohort* | *1936-1945* | | | *1946-1955* | | |
|  | **DF** | 71,857 | 2,612 | 974 | 64,771 | 2,208 | 520 |
|  | **D** | 1,957 | 9,351 | 642 | 1,800 | 8,950 | 368 |
| **70-79** | *Birth cohort* | *1926-1935* | | | *1936-1945* | | |
|  | **DF** | 52,426 | 3,067 | 1,524 | 61,201 | 2,615 | 1,125 |
|  | **D** | 1,675 | 10,927 | 1,370 | 1,676 | 9,194 | 840 |
| **80-89** | *Birth cohort* | *1916-1925* | | | *1926-1935* | | |
|  | **DF** | 20,284 | 2,110 | 1,390 | 33,059 | 2,508 | 1,389 |
|  | **D** | 861 | 8,566 | 1,873 | 1,203 | 9,322 | 1,514 |

**Supplementary Table S4.** Partial cohort life expectancy by two estimation methods

Panel A. Population life expectancies directly estimated by total population (Eq.1)

| Age | Cohort | Female partial LE (year) | | | Male partial LE (year) | | |
| --- | --- | --- | --- | --- | --- | --- | --- |
|  |  | DF | D | Total | DF | D | Total |
| 60-69 | Early | 8.18 (8.07, 8.28) | 1.31 (1.22, 1.40) | 9.48 (9.43, 9.53) | 8.22 (8.11, 8.34) | 1.02 (0.92, 1.10) | 9.24 (9.17, 9.31) |
|  | Later | 8.33 (8.24, 8.42) | 1.27 (1.18, 1.35) | 9.60 (9.56, 9.65) | 8.23 (8.13, 8.35) | 1.18 (1.10, 1.28) | 9.42 (9.35, 9.48) |
| 70-79 | Early | 7.24 (7.13, 7.36) | 1.68 (1.58, 1.78) | 8.92 (8.85, 8.99) | 7.06 (6.92, 7.20) | 1.35 (1.26, 1.43) | 8.41 (8.31, 8.51) |
|  | Later | 7.40 (7.29, 7.51) | 1.61 (1.54, 1.70) | 9.01 (8.94, 9.08) | 7.41 (7.27, 7.52) | 1.29 (1.21, 1.39) | 8.70 (8.60, 8.78) |
| 80-89 | Early | 4.90 (4.76, 5.04) | 2.47 (2.36, 2.59) | 7.37 (7.26, 7.49) | 5.00 (4.81, 5.19) | 1.73 (1.61, 1.84) | 6.73 (6.57, 6.90) |
|  | Later | 5.25 (5.10, 5.39) | 2.33 (2.23, 2.45) | 7.58 (7.47, 7.69) | 4.97 (4.81, 5.13) | 1.87 (1.77, 1.99) | 6.84 (6.70, 7.00) |

Panel B. Population life expectancies aggregate from sub-population as in Eq.2

| Age | Cohort | Female partial LE (year) | | | Male partial LE (year) | | |
| --- | --- | --- | --- | --- | --- | --- | --- |
|  |  | DF | D | Total | DF | D | Total |
| 60-69 | Early | 8.16 (8.06, 8.27) | 1.31 (1.22, 1.40) | 9.48 (9.42, 9.52) | 8.22 (8.10, 8.33) | 1.02 (0.94, 1.11) | 9.24 (9.17, 9.32) |
|  | Later | 8.34 (8.24, 8.45) | 1.26 (1.17, 1.35) | 9.60 (9.56, 9.65) | 8.23 (8.12, 8.34) | 1.19 (1.09, 1.27) | 9.41 (9.35, 9.47) |
| 70-79 | Early | 7.23 (7.12, 7.33) | 1.69 (1.59, 1.78) | 8.92 (8.84, 8.99) | 7.07 (6.93, 7.19) | 1.34 (1.26, 1.44) | 8.41 (8.32, 8.50) |
|  | Later | 7.41 (7.29, 7.52) | 1.61 (1.52, 1.70) | 9.02 (8.95, 9.08) | 7.41 (7.27, 7.52) | 1.29 (1.20, 1.38) | 8.70 (8.61, 8.78) |
| 80-89 | Early | 4.90 (4.75, 5.05) | 2.47 (2.36, 2.58) | 7.37 (7.24, 7.50) | 4.99 (4.81, 5.17) | 1.73 (1.62, 1.84) | 6.72 (6.56, 6.87) |
|  | Later | 5.26 (5.12, 5.40) | 2.33 (2.22, 2.44) | 7.59 (7.49, 7.70) | 4.96 (4.83, 5.10) | 1.88 (1.77, 1.98) | 6.84 (6.71, 6.96) |

*Note:* Values in the parentheses represent 95% CIs.

*Source*: Authors’ calculation based on HRS (22)
